# Preservation of the optic radiations based on comparative analysis of diffusion tensor imaging tractography and anatomical dissection

**DOI:** 10.3389/fnana.2015.00096

**Published:** 2015-08-04

**Authors:** Roland P. Nooij, Eelco W. Hoving, Arjen L. J. van Hulzen, Frans W. Cornelissen, Remco J. Renken

**Affiliations:** ^1^Department of Neurosurgery, University Medical Center GroningenGroningen, Netherlands; ^2^Department of Radiology, University Medical Center GroningenGroningen, Netherlands; ^3^Laboratory of Experimental Ophthalmology, Neuroimaging Center Groningen, University Medical Center GroningenGroningen, Netherlands

**Keywords:** optic radiations, anatomy, *Safety Zone*, diffusion tensor imaging

## Abstract

**Background:** Visualization of the precise course of the visual pathways is relevant to prevent damage that may inflict visual field deficits during neurosurgical resections. In particular the optic radiations (OR) are susceptible to such damage during neurosurgery. Cortical pathways can be mapped *in vivo*, by using Diffusion Tensor Imaging (DTI). Visualization of these pathways would be potentially helpful to prevent neurosurgical visual morbidity. In this study an anatomical dissection of the visual pathways was compared to DTI fiber tractography (DTI-FT) data of four human brains. The feasibility of a definition of a *Safety Zone* is investigated.

**Methods:** Four adult brains were dissected using Klingler's fiber dissection method, which allowed preparation of the OR. Measurements before and after dissection were used to establish distances from the cortex to the OR. DTI-scans were also obtained from these brains to determine the same distances.

**Results:** Measurements from specific landmark points on the cortex to the lateral border of the OR were performed in four brains. Analysis through DTI tractography corresponded with the dissection results. Based on the combined results of both dissection and DTI-FT, we defined a quantitative surgical *Safety Zone* with respect to various anatomical landmarks (in particular the ventricle system).

**Conclusion:** We conclude that there is a good correlation between the visualizations of the optic pathways based on dissection and DTI. Furthermore, we conclude that defining a neurosurgical *Safety Zone* which could preserve the integrity of the OR during surgery, based on the combination of DTI-FT images and dissection is feasible.

## Introduction

The anatomical course of axons within the human brain has been initially studied using post-mortem dissections and histological investigations (Forel, 1877; Dejerine, 1901; Riley, 1953; Talairach, 1957; Lemaire et al., 2011). Identification of specific pathways (e.g., corticospinal tract, the optic radiations, and the acoustic radiations) has been feasible in the human brain (Forel, 1877; Dejerine, 1901; Riley, 1953; Talairach, 1957; Axer et al., 2000; Lemaire et al., 2011). Knowledge of the course of these pathways is of great relevance in neurosurgery (Lemaire et al., 2011). Visualization of these pathways could help prevent neurosurgical damage during resections, in particular by establishing a safety zone that should be respected during neurosurgery.

**Figure 1:**
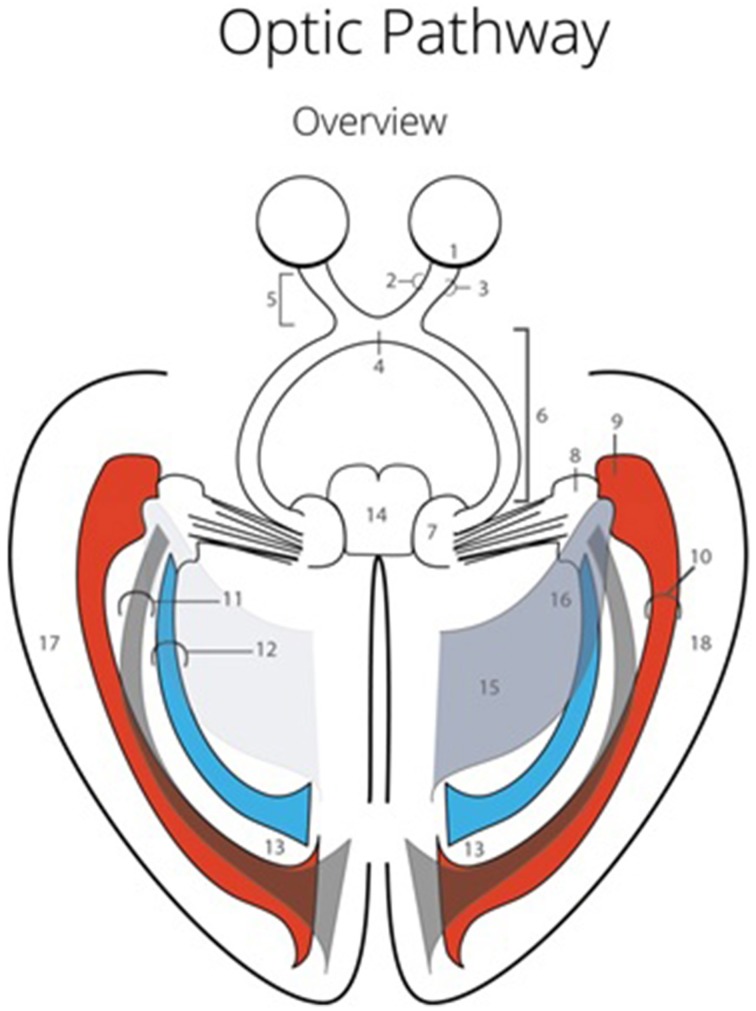
**Optic Pathway**. 1, Retina; 2, Nasal retinal nerve fibers; 3, Temporal retinal nerve fibers; 4, Optic chiasm; 5, Optic nerves; 6, Optic tract; 7, Colliculus superior (dextra); 8, Lateral geniculate body (dextra); 9, Meyer's loop; 10, Anterior bundle of the optic radiations; 11, Central bundle of the optic radiations; 12, Dorsal bundle the optic radiations; 13, Visual cortex (occipital lobe); 14, Mesencephalon; 15, Trigonum (or atrium) of the ventricle system; 16, Temporal horn of the ventricle system; 17, Left temporal lobe; 18, Right temporal lobe.

The visual pathways have a horizontal and elongated course through the entire brain, which increases the chance of damaging these pathways (Hofer et al., 2010) (see Figure 1). The optic radiations (OR) cannot be identified using the standard view through a microscope (Hofer et al., 2010). This could lead to damage to the OR during neurosurgical resections (Ebeling and Reulen, 1988; Krolak-Salmon et al., 2000; Sincoff et al., 2004; Yasargil et al., 2004; Powell et al., 2005; Rubino et al., 2005; Taoka et al., 2005; Choi et al., 2006; Peltier et al., 2006; Sherbondy et al., 2008). Surgical morbidity due to damage of the OR, is quite common (Hughes et al., 1999; Krolak-Salmon et al., 2000; Hofer et al., 2010). This damage often results in a visual field defect, which correlates with the affected bundle of the OR (Sherbondy et al., 2008). Furthermore, there is a significant inter-patient variability with regards to the exact course of the OR (Ebeling and Reulen, 1988; Sherbondy et al., 2008). Due to this individual variation, particularly pronounced at Meyer's loop (Ebeling and Reulen, 1988; Sherbondy et al., 2008), it is highly preferable to map the exact anatomy of the OR in individual patients(Ebeling and Reulen, 1988; Krolak-Salmon et al., 2000; Sincoff et al., 2004; Yasargil et al., 2004; Powell et al., 2005; Rubino et al., 2005; Taoka et al., 2005; Choi et al., 2006; Kikuta et al., 2006; Peltier et al., 2006; Okada et al., 2007; Sherbondy et al., 2008).

Pathways can be visualized *in vivo* using diffusion tensor imaging (DTI) (Chenevert et al., 1990; Douek et al., 1991; Basser et al., 1994; Coremans et al., 1994; Nakada and Matsuzawa, 1995; Axer et al., 2000; Mori and Zhang, 2006; Lemaire et al., 2011). This technique relies on the anisotropic diffusion of water molecules in and alongside nerve axons, which result in a raw image of the pathways that represents their anatomy (Basser et al., 1994; Mori and Zhang, 2006). The DTI is an imaging method which is based on a mathematical model which describes the three-dimensional diffusion in different axes around fiber bundles (Potgieser et al., 2014). This means that the DTI-fiber tractography (DTI-FT) method is an interpretation of the organization of fiber bundles, based on the preferential diffusion of water molecules (Potgieser et al., 2014).

Fiber tractography performed on DTI-scans visualizes specific white matter pathways in the brain based on selected algorithms (e.g., continuous tracking, a deterministic FT method) (Chenevert et al., 1990; Coremans et al., 1994; Mori and Zhang, 2006; Sherbondy et al., 2008; Hofer et al., 2010; Lemaire et al., 2011). Both volumetric and quantitative measurements can be performed on the pathway visualized through the DTI-FT method (Chenevert et al., 1990; Coremans et al., 1994; Mori and Zhang, 2006; Sherbondy et al., 2008; Hofer et al., 2010; Lemaire et al., 2011). Anatomical studies have contributed much to the knowledge of the course of pathways in the brain (Forel, 1877; Dejerine, 1901; Riley, 1953; Talairach, 1957; Lemaire et al., 2011). The DTI-FT technique allows mapping of the pathways *in vivo* (Chenevert et al., 1990; Coremans et al., 1994; Mori and Zhang, 2006; Sherbondy et al., 2008). Combining these two methods would potentially grant reliable insight in the course of the OR in the human brain (Ebeling and Reulen, 1988; Krolak-Salmon et al., 2000; Sincoff et al., 2004; Yasargil et al., 2004; Powell et al., 2005; Rubino et al., 2005; Taoka et al., 2005; Choi et al., 2006; Kikuta et al., 2006; Peltier et al., 2006; Lemaire et al., 2007; Okada et al., 2007; Sherbondy et al., 2008), and validate the *in vivo* images (Sherbondy et al., 2008).

This study focuses on visualization of the course and key points of the OR through both anatomical dissection and DTI-FT. Combining both DTI-FT and anatomical dissection of the OR, the degree of correspondence could be established between the results of these different techniques. This correlation was firstly investigated, after which it was used to validate the accuracy of the DTI-FT method. The visualization of the OR in four human brains using these two techniques provided the basis of defining a surgical *Safety Zone* with quantitative measurements related to anatomical landmarks (i.e., the ventricle system).

## Material and methods

### Brains and preparation

The brains that were used in this study were post mortem brains: ♂79 years, ♂82 years, ♂80 years, ♂76 years with no pathology of the central nervous system. In accordance with ethical standards post mortem material was obtained from donors willing to donate their body for purpose of science through the Dissection hall of the University Medical Center Groningen.

All brains underwent preparation according to Klingler's fiber dissection method (Ebeling and Reulen, 1988; Sherbondy et al., 2008). Klingler's fiber dissection method makes it possible to dissect fibers tracts after a brain is held on a temperature of −15 to −20 degree centigrade for approximately 2 weeks. After this process, the consistency of the brain makes it possible to dissect fiber tracts. The freezing process causes a volumetric change, due to the fact that water expands for 10% (also spreading them somewhat apart from each other which makes them easier to dissect).

Four formalin-fixed brains were held on a temperature of −15 to −20 degree centigrade for 2 weeks after the formalin was washed out, after which dissection started through Klingler's fiber dissection method (Ebeling and Reulen, 1988; Sherbondy et al., 2008).

### Measurements pre-dissection

**Figure 2:**
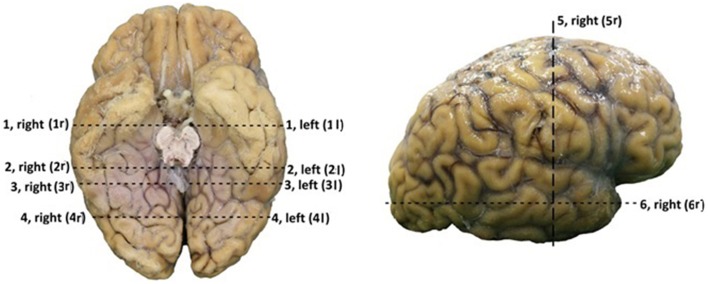
**Points of measurements on the exterior part of the brain, one from a caudal perspective (left) and one from a lateral perspective (right)**. Both hemispheres were measured, resulting in a total of 12 measurements. 1, rostral part of brain stem to superior temporal gyrus; 2, caudal part of brain stem to middle temporal gyrus; 3, splenium of the corpus callosum to temporal cortex; 4, 25 mm caudal of splenium of the corpus callosum to temporal cortex; 5, central sulcus to inferior temporal gyrus; 6, temporal pole to occipital pole.

Before the dissection started, several external specific landmark points on the cortex were identified in order to calculate the distance to the OR (Figure 2). The results of these measurements are shown in Table A1.

### Preparation of the optic radiations

Dissection on the post mortem brains (which were preserved through the process of formalin fixation, after which they were held on −15 to −20 degree centigrade for 2 weeks) could start after they were washed with warm water for < 5 min. This process caused for the time difference between DTI acquisition through MRI scans, which needed to be performed before the freezing process, and dissection.

**Figure 3:**
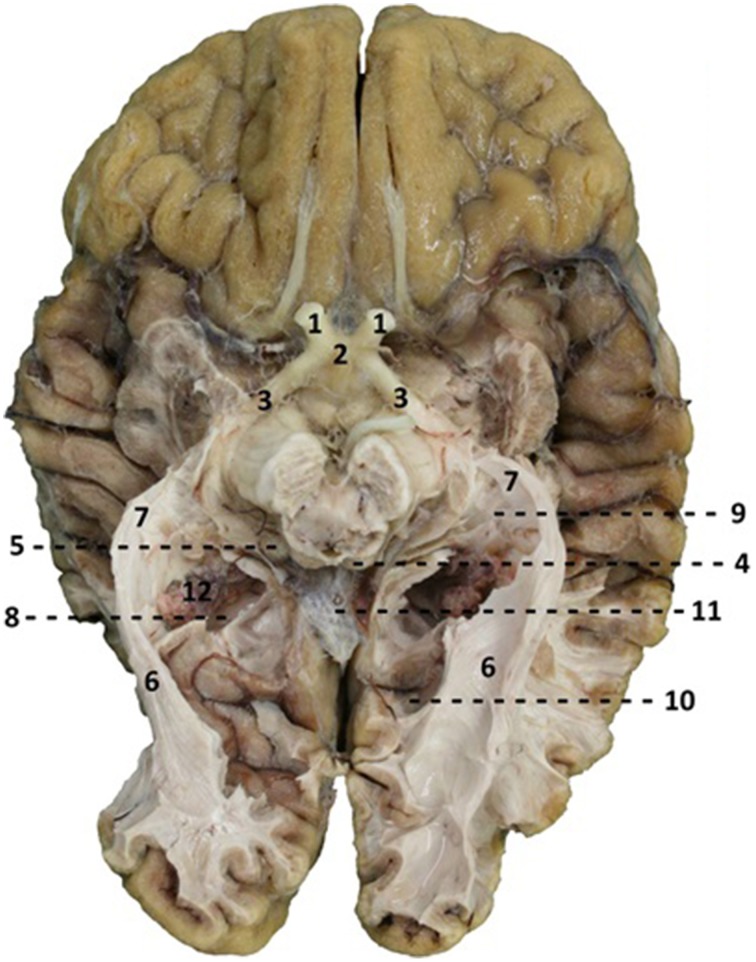
**Caudal view of a brain after dissection**. 1, optic nerve (NII); 2, optic chiasm; 3, optic tract; 4, colliculi superior; 5, lateral geniculate body; 6, optic radiations; 7, Meyer's loop; 8, trigonum of ventricles; 9, temporal horn of ventricles; 10, occipital horn of the ventricles; 11, corpus callosum, splenium.

The dissection of the brains started at the inferior surface of both the occipital and the temporal lobe. After thawing, the cortex of these lobes could be dissected on the inferior surface. Using this technique, the course of the optic tract and its key structures could be identified. Through this process the optic tract became better visible. This allowed following the fibers of the OR through the visualization of key structures of the optic tract. The fibers of the OR could be identified by visualizing the optic chiasm, the optic tract circumventing the mesencephalon to the colliculi superioris and the lateral geniculate body (LGB). The LGB could be identified through the removal of the parahippocampal gyrus and by following the brachium colliculi superioris, originating from the colliculi superioris. Subsequently, the fibers originating from the LGB (the OR) underwent preparation using the ventricle system as orientation due to their close relationship with the OR (the OR is situated alongside the ventricles) (Ebeling and Reulen, 1988; Rubino et al., 2005; Peltier et al., 2006) (see Figure 3). Furthermore, the dissection of the inferior surface of the occipital and temporal lobe allows a view (caudal) on the ventricle system, specifically the temporal and occipital horns which have an important relation with the OR. When the temporal horn is exposed due to the dissection from the inferior surface of the temporal lobe, the roof of the temporal horn is revealed which is an important orientation point with respect to the OR.

### Measurements post-dissection

**Figure 4:**
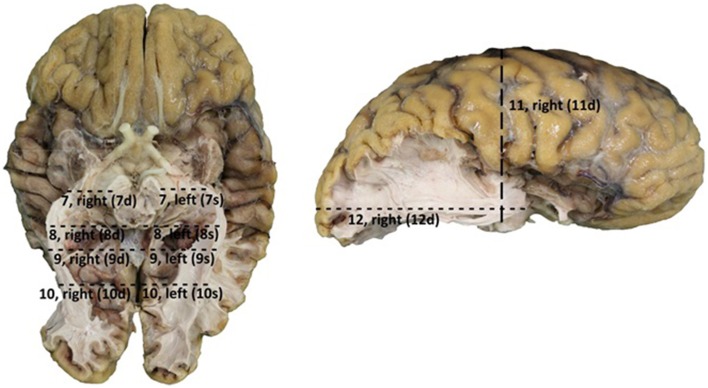
**Points of measurement on the brain after dissection, 1 from a caudal perspective (left) and 1 from a lateral perspective (right)**. Both hemispheres were measured, resulting in a total of 12 measurements. 7, rostral part ofbrain stem to OR; 8, caudal part of brain stem to outer border OR; 9, splenium of the corpus callosum to outer border OR; 10, 25 mm caudal of splenium of the corpus callosum to outer border OR; 11, central sulcus to lower border of the OR; 12, occipital pole to anterior part of the OR (Meyer's loop).

After the dissection was completed, additional measurements were performed in order to determine the distance between the OR and the earlier mentioned landmark points on the cortex. The length of the OR was also measured (see Figure 4). The results of these measurements are presented in Table A2.

The results from Table A2 needed to be subtracted from the results of Table A1. This subtraction represents the distance between the lateral border of the OR and the predetermined landmarks on the cortex. These results are shown in Table A3. The results from Table A3 were averaged and the standard deviation was determined using the data from this table. The data from both hemispheres of each brain was added up and divided with eight, determining the mean distance from the OR to the predetermined landmarks on the cortex in a hemisphere. The deviation of each data point was determined and squared. By adding these results up and dividing them with eight, the variance was determined. The square root of this variance was determined as the population standard deviation for that specific data point. The aim of this statistical analysis is to determine the dimensions of the lateral border of the OR with respects to the predetermined landmarks on the cortex in a hemisphere and not specifically the left or right hemisphere.

Measurements between the lateral border of the OR and the lateral border of the ventricle system were also taken. This represents the lateral extension of the OR at predetermined landmarks of the ventricle system and was measured at point 8, 9, and 10 (Figure 4). These results were averaged and their standard deviation was determined the same way as was done for the results regarding the distance from the lateral border of the OR to the predetermined landmarks on the cortex. These results respectively represent the lateral extension of the OR at the temporal horn, the trigonum and the occipital horn of the ventricle system. The ventricle system was used as an anatomical landmark in order to define a *Safety Zone*.

### MRI

Three post mortem brains were scanned on a 3.0 Tesla MRI scanner at the Neuroimaging Center in Groningen (Philips Intera; Eindhoven, The Netherlands). To the best of our knowledge, DTI-FT has not yet been performed on *ex vivo* human post mortem brains. An attempt has been made to perform a DTI scan on a post mortem frozen brain as a pilot, which was unsuccessful. Although there was no usable DTI data of this brain, dissection has been performed in order to gain dissection data of this brain. DTI scans of the other three post mortem brains (non-frozen; held in a container with saline solution; possibility for diffusion of water in the post mortem brain) were made, which were of adequate quality to perform tractography on.

High resolution T_2_-weighted scans were acquired using the WIP DTI/s/60dir/APP SENSE protocol with a 90 degree flip angle; matrix size 128 × 128 mm and a field of view of 240 × 140 × 240 mm yielding 70 slices and a voxel dimension of 1.875 × 1.875 × 2 mm. All brains could be processed properly using the *Diffusion Toolkit* of the *Trackvis* program (Ruopeng Wang, Van J. Wedeen, www.TrackVis.org, Martinos center for Biomedical Imaging, Massachusetts General Hospital). Brains that needed correction with regards to the axes (X-, Y-, and Z-axis) due to their position within the MRI scanner were performed by using the *Diffusion Toolkit*. This way, fiber tractography could be performed on the scanned brains.

### Processing of MRI data: Region of interest (ROI) specifications

In order to adequately perform fiber tractography, the axes in which the post mortem brain was scanned needed to be adjusted. They were not in an anatomical position due to the fact that the post mortem brains were held in a container with saline solution (for example, the inferior surface of the brain was orientated superiorly). This was necessary because of the importance of the orientation of the brain in three axes with regards to its fiber directions and color coding of those fibers. The necessary inversions with regards to the axes could be performed with the *Diffusion Toolkit* program. They were executed by addressing the tab *X-, Y-, Z-axis*. By inverting the axes (Z-axis to Y-axis, Z-axis to X-axis, X-axis to Y-axis, X-axis to Z-axis, Y-axis to X-axis, Y-axis to Z-axis), the orientation of the brain (and its fibers) within the *TrackVis* program was “anatomically” correct after which fiber tractography could start. The fast continuous tracking algorithm (FACT) was used as the fiber tracking algorithm. A deterministic method of fiber tracking (i.e., FACT) allows for quick tractography based on ROIs placed in the DTI scan by the observer.

To track the course of the OR in *TrackVis*, spherical regions of interest (ROI) were used. The ROIs were placed on known anatomical key structures of the OR which are identifiable on the T_2_-weighted DTI scans. These structures were: the LGB, the lateral wall of the temporal horn, trigonum, and occipital horn of the ventricle system and the visual cortex.

**Figure 5:**
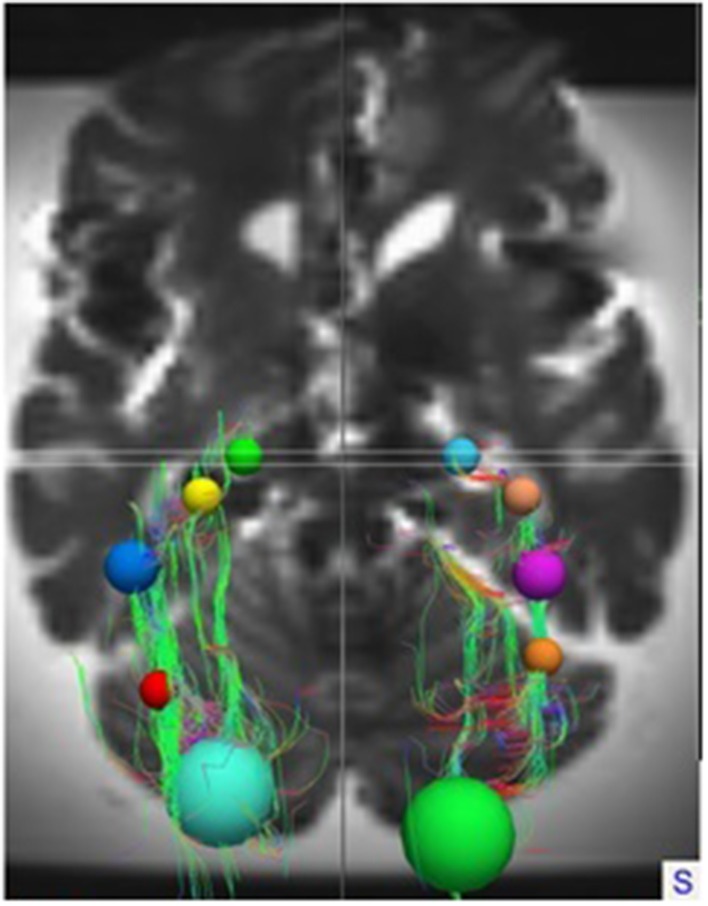
**Superior view on the entire brain showing the ROIs and the OR as identified by TrackVis**. For orientation, the ROIs used in the right hemisphere are: Light Blue, LGB ROI; Light Brown, Temporal horn ROI; Purple, Trigonum of ventricles ROI; Orange, Occipital horn ROI; Green, Visual cortex ROI.

**Figure 6:**
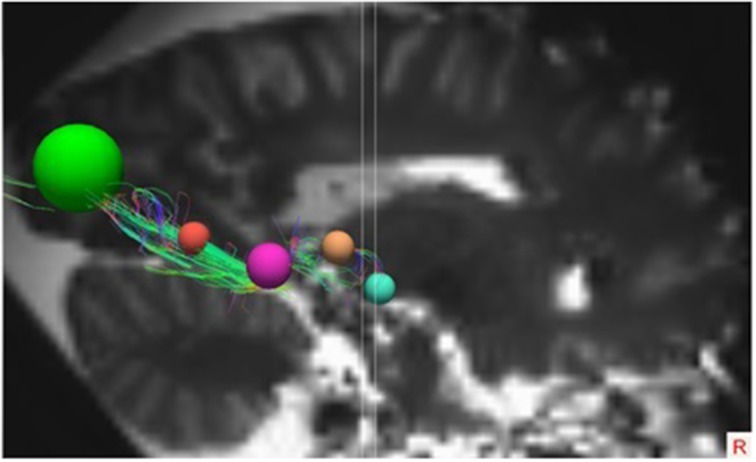
**Lateral view of the right hemisphere with the specific ROIs showing the OR**. The ROIs in this right hemisphere are the same ROIs used in the right hemisphere in Figure 5.

These ROIs were defined for both hemispheres in all brains. In each individual brain, the ROIs were manually placed on the earlier mentioned anatomical key structures in order to track the OR from the LGB to the visual cortex. This deterministic method of fiber tractography resulted in the visualization of the OR. Figures 5, 6 show the placement of these ROIs in one of the brains as well as the fibers resulting from the tractography.

Measurements of the lateral extension of the OR with regards to the ventricle system and the length of the OR could be performed. These results were averaged and their standard deviation was determined the same way as was done with the results from the dissection.

## Statement of ethical approval

The research protocol was approved by the Institutional Review Boards of the University Medical Center Groningen as a scientific research project and was conducted according to the principles expressed in the Declaration of Helsinki. The use of post mortem material in this study was approved by the medical faculty of the University Medical Center Groningen.

## Results

### Dissection

**Figure 7:**
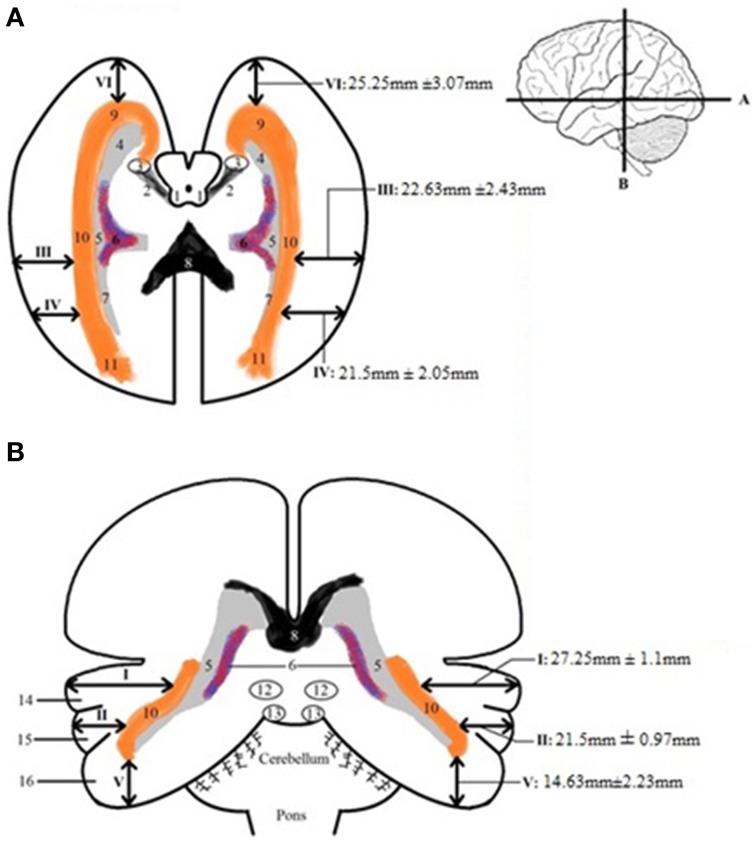
**Schematic depicture of the dimension of the OR in respect to the cortex**. **(A)** Transversal slice depicting results. **(B)** Coronal slice depicting results. The measurements that are depicted are the averages (with the mean standard deviations) from the results in Table A4. 1, colliculi superior; 2, brachium colliculi superiors; 3, lateral geniculate body; 4, temporal horn of the ventricles; 5, trigonum; 6, choroid plexus of lateral ventricles; 7, occipital horn of the ventricles; 8, corpus callosum, splenium; 9, OR, Meyer's loop; 10, OR; 11, fibers of OR end up in visual cortex; 12, colliculi superior; 13, colliculi inferior; 14, superior temporal gyrus; 15, middle temporal gyrus; 16, inferior temporal gyrus.

Table A3 shows the calculated distance from the predetermined landmarks on the cortex (see Figure 2) to the lateral border of the OR and the measured length of the OR. The results were averaged and the standard deviation was determined for each data point. These results can be seen in Table A4 and are shown in Figure 7, which visualizes the dimensions of the OR with respect to the predetermined landmarks on the cortex.

By averaging the results of Table A3, the dimensions of the OR with regards to the cortex were established. This can be seen in Figure 7. These results correspond with previous anatomical studies (Ebeling and Reulen, 1988; Choi et al., 2006; Peltier et al., 2006; Sherbondy et al., 2008).

After the dissection it was possible to measure the lateral extension of the OR. This was completed by measuring the distance from the lateral borders of the OR to specific points of the ventricular system (i.e., the lateral border of the temporal horn, the trigonum and the occipital horn). The results of these measurements are shown in Table A5. These results correspond with previous anatomical studies (Ebeling and Reulen, 1988; Choi et al., 2006; Peltier et al., 2006; Sherbondy et al., 2008).

**Figure 8:**
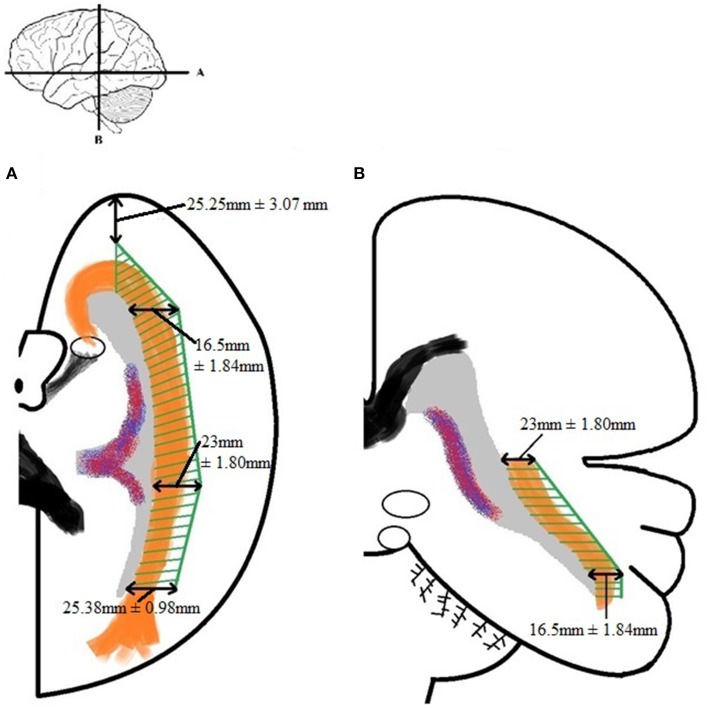
**Schematic depicture of the *Safety Zone* for the OR established through the dissection results**. **(A)** Transversal slice depicting results. **(B)** Coronal slice depicting results. The *Safety Zone* (green) envelops the OR (orange), as to ensure its integrity. The dimensions of the *Safety Zone* are equal in both hemispheres. Therefore only one hemisphere is depicted in this figure.

By using the results from Table A5 and the smallest distance between the temporal pole and Meyer's loop (Table A4, measurement *VI*) as cut-off value, an attempt has been made to determine a *Safety Zone* using the ventricle system as a landmark. This method has been used to ensure the integrity of the OR within the limited population available in this study. These results are shown in Figure 8.

### DTI-FT

**Figure 9:**
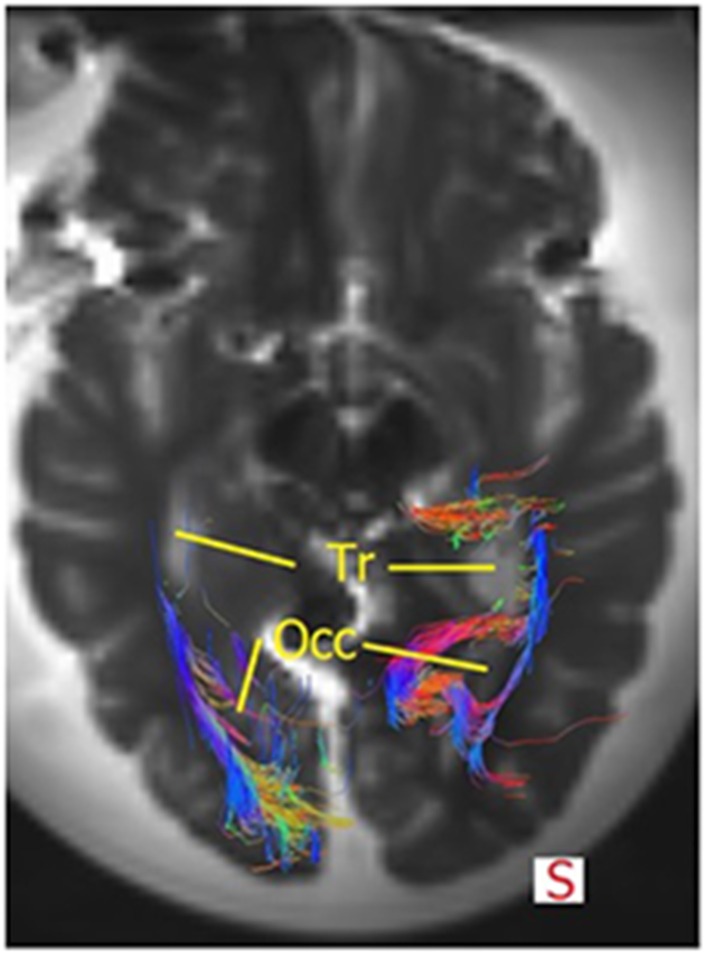
**Superior view of the brain and the OR**. The fibers course alongside the trigonum (=*Tr*) and the occipital hom (=*Occ*). Some fibers of the right OR also traverse medially at the occipital horn to also end up in the visual cortex. Mind that this section does not show all fibers of the OR or the temporal horn (see Figure 10).

**Figure 10:**
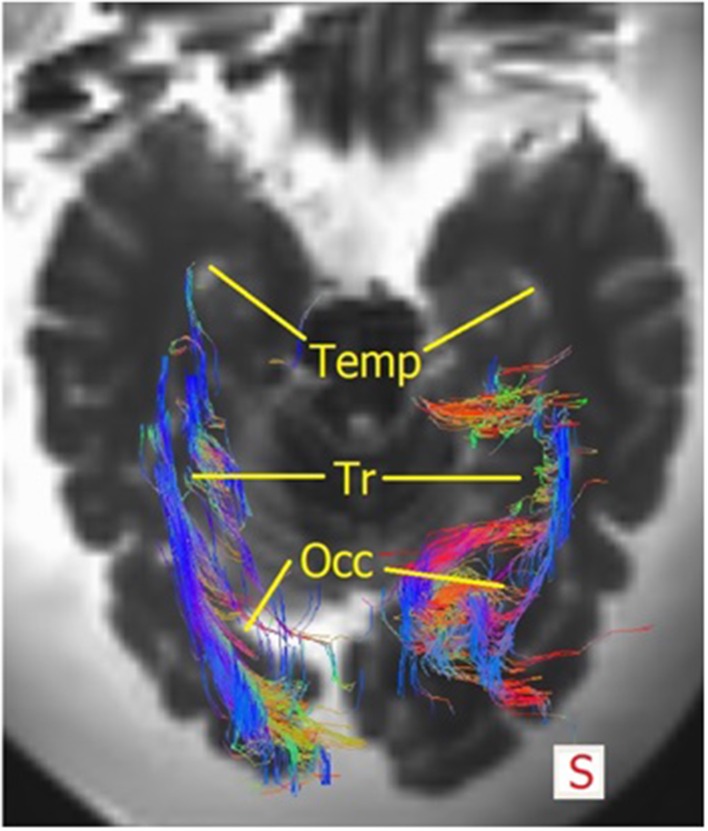
**Superior view of the brain with the fibers of the OR**. The right OR traverse laterally over the temporal horn (=*Temp*), then courses alongside the trigonum (=*Tr*) after which a portion of the fibers traverse medially at the occipital hom (=*Occ*) and some course laterally alongside the occipital horn to end up in the visual cortex. Mind that this section is lower than Figure 9, therefore not evidently showing the trigonum/occipital horn.

The OR was analyzed using the *TrackVis* program, which allowed for measurements of the length and the lateral extension of the OR. The relationship of the OR with the ventricles became evident, as can be seen in Figures 9, 10. The OR traverses laterally over the temporal horn of the ventricles (Figure 10) after which it continues alongside the trigonum of the ventricles and the occipital horn of the ventricles (Figures 9, 10) to end up in the visual cortex.

The lateral extension of the OR was measured at the temporal horn, trigonum, and the occipital horn of the ventricle system. This was done to confirm the *Safety Zone* established based on the dissection data (Figure 8) with the DTI-FT data. Table A6 shows the lateral extension and length of the OR. These results correspond with previous anatomical studies (Ebeling and Reulen, 1988; Choi et al., 2006; Peltier et al., 2006; Sherbondy et al., 2008).

### Comparative analysis of the dissection and DTI-data

**Figure 11:**
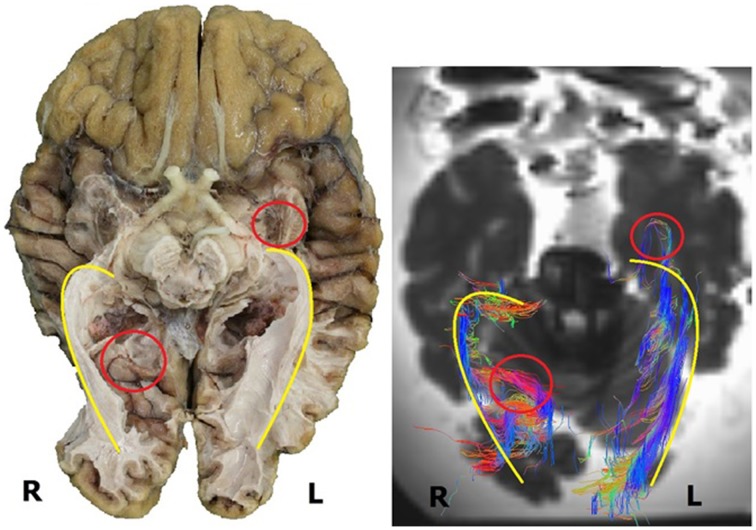
**Inferior view of a human brain, both dissection (left) and DTI-scan (right)**. Several similarities and differences can be deduced from this image. The similarities in the general course of the OR are indicated by the yellow line. The differences are indicated by the red circle, i.e., the difference in the extension of Meyer's loop (left) and the difference in the fibers at the occipital horn (right). R, right; L, left.

As can be observed in Figure 11, the general course of the OR and its dimensions as established by the dissection results and the DTI-scans correspond with each other. These similarities seem to be common in the sample used in this study and Figure 11 (of one brain) has been used to clarify this. In this example, the right OR seems to be slightly more curved than the left OR. This can be seen both in the dissection and DTI-FT results (yellow line).

However, Figure 11 (as an example for the whole sample used in this study) also shows the difference in detail between both methods with respect to the tracking of all the fibers. In the left hemisphere, the DTI-FT data shows a Meyer's loop which extends far more anteriorly than was anticipated on the basis of dissection results (red circle). This is illustrative for how delicate and vulnerable the Meyer's loop fibers are. Furthermore the fibers of the OR which traverse medially over the occipital horn on the right hemisphere are not shown in the dissection results (red circle), while these were revealed through the DTI-FT data. This specific difference in detail could be caused by the balance between the preservation and dissection of fibers in order to adequately visualize the OR.

### Safety zone

By examining the results with respect to the lateral extension of the OR, both the dissection and DTI-FT data, a *Safety Zone* which would ensure its integrity based on both methods can be established.

**Figure 12:**
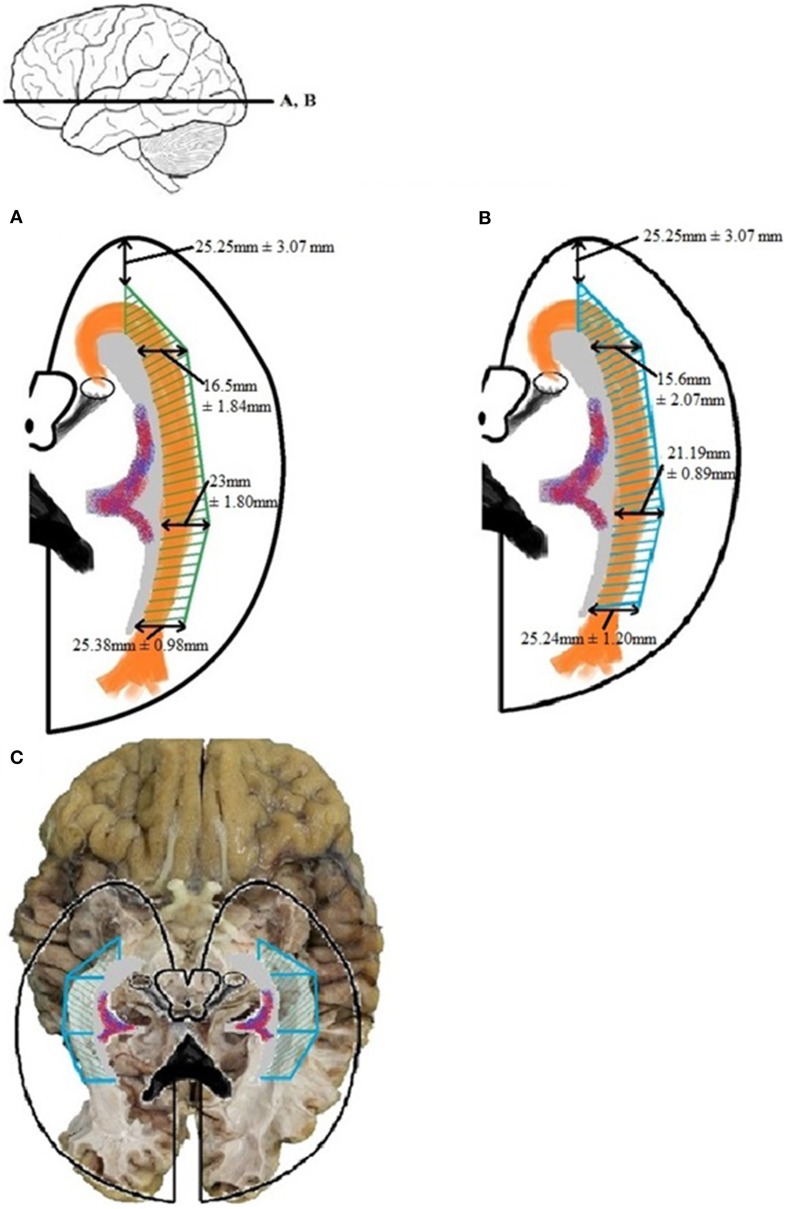
**The *Safety Zone* which was established through the results of dissection (A) has been compared with the results from the DTI-FT (B), originating from Table A6**. The *Safety Zone* composed based on the dissection results envelops *Safety Zone* based on the DTI-FT results, thereby confirming the dimensions. The confirmed *Safety Zone* (blue) has been placed over a photograph of the dissection **(C)**. The OR is enveloped by the *Safety Zone*.

Establishing a *Safety Zone*—which would ensure the integrity of the OR during surgery—can be accomplished by using the dissection measurements of Tables A4 and A5 as cut-off values. These results were confirmed by the DTI-FT data. The measurements needed for this are the lateral extension of the OR at the predetermined locations of the ventricle system (given in Table A6). The confirmed *Safety Zone* based on both dissection and DTI-FT data is shown in Figure 12. Unfortunately, the distance from the temporal pole to Meyer's loop could not be calculated using *TrackVis*. Therefore, the result from measurement *VI* of Table A4 has been applied for this distance in Figure 12.

## Discussion

Our main conclusion is that the OR course and dimensions as established on the basis of DTI-FT data and the dissection results are very similar. Therefore, we infer that it is feasible to establish a *Safety Zone* for use during a neurosurgical resection. Additionally, the quality of both separate techniques (DTI-FT, dissection) with regards to tracking cortical pathways could be evaluated.

### Implication safety zone

The *Safety Zone* was established based on a combination of dissection and DTI-FT results, which we believe strengthens its validity. Moreover, the results used to establish it correspond with those of previous studies (Ebeling and Reulen, 1988; Choi et al., 2006; Peltier et al., 2006; Sherbondy et al., 2008). The *Safety Zone* which we have established in this study could be used to determine—in general—if the OR is at risk during a neurosurgical resection (Ebeling and Reulen, 1988; Krolak-Salmon et al., 2000; Sincoff et al., 2004; Yasargil et al., 2004; Powell et al., 2005; Rubino et al., 2005; Taoka et al., 2005; Choi et al., 2006; Peltier et al., 2006; Sherbondy et al., 2008). It could therefore be a tool in order to preserve the OR during neurosurgery. By identifying key structures of the OR which are recognizable on T_2_-weighted DTI scans within the *Safety Zone*, it can also function as an indicator to determine where ROIs need to be placed in order to isolate the OR with fiber tractography.

Nevertheless, applying the proposed *Safety Zone* should be done with caution, as the population on which it was based was limited. Also, aging and pathological processes could change or distort the course of the fibers of the OR as a result of which the present *Safety Zone may* be too strict. Hence, the here defined *Safety Zone* should only be considered as providing the anatomical margins for localizing the OR.

### Potential of DTI

Surgical visual morbidity has been reported to be common (Hughes et al., 1999; Krolak-Salmon et al., 2000; Hofer et al., 2010). By applying the method of DTI-FT prior to neurosurgical resections, insight can be given in the anatomical course of the OR in an individual in order to prevent injury. In order to benefit optimally from presurgical planning, a DTI-FT could be performed to track the fibers of the OR and determine their course. This could lead to an adjusted approach in order to protect the OR. Due to the individual variation of the OR, which is mostly found at Meyer's loop (Ebeling and Reulen, 1988; Sherbondy et al., 2008), it is highly preferable to map the exact anatomy of individual patients (Ebeling and Reulen, 1988; Krolak-Salmon et al., 2000; Sincoff et al., 2004; Yasargil et al., 2004; Powell et al., 2005; Rubino et al., 2005; Taoka et al., 2005; Choi et al., 2006; Kikuta et al., 2006; Peltier et al., 2006; Okada et al., 2007; Sherbondy et al., 2008). Therefore, accurately visualizing the OR *in vivo* through DTI-FT has potentially enormous benefits for neurosurgical patients (Chenevert et al., 1990; Coremans et al., 1994; Mori and Zhang, 2006; Sherbondy et al., 2008) as it provides viable and applicable information in planning the approach of neurosurgical resections. Nevertheless, during any neurosurgical procedure the pre-operative individual delineation of the OR may be compromised by brain shift. Other factors that may affect the localization and visualization of the OR by DTI-FT are brain edema and the type of lesion.

Unfortunately, up until now and to the best of our knowledge there is no validation method for the fibers tracked using the deterministic DTI-FT method, as is being used in this study. In addition, the use of manually placed ROIs in the deterministic method to identify pathways may lead to unwanted bias. Meaning that the ROIs are placed in such a manner, that the pathway resembles the anatomy as is expected from anatomical literature. Identifying key structures in the course of the OR in order to successfully isolate the OR in fiber tractography could potentially grant benefits. Suggested structures which are easily recognized on T_2_-weighted DTI scans on which ROIs could be placed in order to isolate the OR with a deterministic method of fiber tracking include: the LGB, the lateral wall of the temporal horn, trigonum, and occipital horn of the ventricle system and the visual cortex. This modality (fiber tractography based on a predetermined set of recognizable structures of the OR on T_2_-weighted scans) could grant benefits for the clinicians in the form of relative quick fiber tractography to isolate the OR in DTI scans.

Still, we believe that the DTI-FT method creates potential benefits with regards to presurgical planning and mapping of the OR (Ebeling and Reulen, 1988; Krolak-Salmon et al., 2000; Sincoff et al., 2004; Yasargil et al., 2004; Powell et al., 2005; Rubino et al., 2005; Taoka et al., 2005; Choi et al., 2006; Kikuta et al., 2006; Peltier et al., 2006; Okada et al., 2007; Sherbondy et al., 2008). Still, it is important to realize that DTI-FT is an imaging method that provides an interpretation of the organization of fiber bundles, based on the preferential diffusion of water molecules. As a result, some intrinsic issues concerning the visualization of the anatomy of the OR should be mentioned, that also became apparent in this study. Meyer's loop of the OR contains a sharp angle which renders it more difficult to track than the rest of the OR. This may cause the DTI-FT method to either miss fibers belonging to the OR or to contribute fibers to the OR which are not part of the optic tract (e.g., uncinate fasiculus or inferior longitudinal fasciculus). This problem is caused by the need to adjust the settings in the fiber tractography program (e.g., the maximum angle of the fibers, the fractional anisotropy, the maximum length of the tracked fibers) in order to visualize the entire optic tract from LGB to visual cortex, including the sharp angle at Meyer's loop. The inter-patient variability in the course of the OR, especially at Meyer's loop (anterior translation, curvature, and angle), may require different settings in every patient for optimal visualization. Unfortunately, to the best of our knowledge, there is no validation method at present for the fibers tracked using the deterministic DTI-FT method that was used in this study. Use of probabilistic DTI-FT, could lead to a more accurate and less biased visualization of the OR, but its much more time-consuming computations could limit its clinical application at present. Another limitation of the deterministic method is the need to manually place ROIs to identify pathways. This may result in placing the ROIs in such a way that the resulting pathway resembles what is expected. This, however, need not necessarily be the correct or complete pathway. We propose a methodology in order to isolate the OR in T_2_-weighted DTI scans with a deterministic method of fiber tractography by suggesting a predetermined set of key structures of the OR which are recognizable where to place ROIs. These are the LGB, the lateral wall of the temporal horn, trigonum and occipital horn of the ventricle system and the visual cortex.

### Dissection method and visualization of fibers

**Figure 13:**
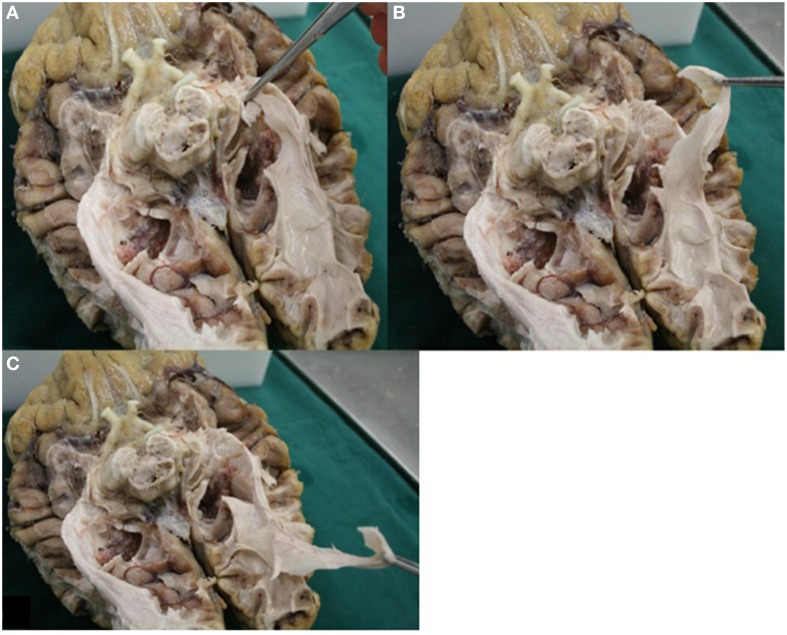
**Image displays the possibility of control in dissection**. Fibers of the OR could be pulled all the way back to the visual cortex. **(A)** Fibers presumably belonging to the OR (Meyer's loop) were grabbed. **(B)** These fibers could be pulled back alongside the lateral wall of the ventricles (in accordance to the known anatomy of the OR). **(C)** Fibers which were grabbed could be pulled all the way back to the visual cortex.

Visualizing fibers with dissection is a viable method to provide insight in the general course of the pathway to which the fibers belong (Forel, 1877; Dejerine, 1901; Riley, 1953; Talairach, 1957; Ebeling and Reulen, 1988; Axer et al., 2000; Peltier et al., 2006). By using the fiber dissection method of Klingler, fibers are easier to follow (Ebeling and Reulen, 1988). With respect to the OR, following the pathway from the optic chiasm to the visual cortex is done by identifying anatomical key structures of the OR. These are the optic tract (which courses along the brainstem at the level of the mesencephalon), the superior colliculi and the LGB. In this manner the fibers of the OR can be identified alongside the ventricle system to the visual cortex (Figure 13). An additional benefit of dissection is the possibility to control if the fibers (which are presumed to belong to the OR) truly belong to the OR. Figure 13 shows how the fibers—which are presumed to belong to the OR—can be pulled all the way back to the visual cortex. This is an anatomical technique to validate whether dissected fibers do in fact belong to the OR.

In order to visualize the entire OR one needs to delicately balance preservation and dissection of fibers. Dissection needs to stop at the lateral border of the OR, even though at this point only a limited visualization of the entirety of the fibers of the OR is realized. Primarily the fibers which run transversally and alongside the lateral border of the ventricles will be visible. Moreover, one should always be aware that fibers which belong to the OR can be accidentally removed during the dissection without even being noticed.

### Future studies

Future dissection studies could increase the number of brains, in order to more accurately determine the course and dimensions of the OR with respect to the ventricle system. This could lead to better insight in the variability of the OR in patients and the two hemispheres. This—in turn—can be used to improve the accuracy of the *Safety Zone*.

Also, the collection of additional *in vivo* data (i.e., DTI-FT) in healthy individuals or patientsis recommended. This way, the variability in the course and dimensions of the OR can be determined more accurately. Again, this can help to further improve the *Safety Zone*. Furthermore, performing preoperative and postoperative DTI-scans complemented with visual field measurements (i.e., static or kinetic perimetry) in a clinical setting could provide useful information on the preservation of the OR and the prevention of postsurgical visual field deficits. Another method to complement the mapping of the OR could be the use of visual evoked potentials (VEPs) during surgery, although the implication of the VEPs is not clear yet (Kim et al., 2013). This way, presurgical planning could be controlled by intra-operative VEPs that test the integrity of the OR (by establishing whether or not the VEPs reach the visual cortex) (Kim et al., 2013).

## Conclusion

We used a combination of dissection and DTI-FT methods to visualize the optic pathways in four human cadaveric adult brains. Both methods were also compared to each other. This provided insight in the advantages and disadvantages of each method for determining the course of the OR.

Based on the combined results, we proposed a neurosurgical *Safety Zone*. This zone can be used for: determining a general zone for preserving the OR during neurosurgery (especially Meyer's loop, due to its inter patient variation), placing ROIs in order to track the OR with DTI-FT and, as an indicator for intra-operative mapping of the OR (e.g., if there is the need to use VEPs for the evaluation of the integrity of the OR). Further research is needed—preferably in a clinical setting—to determine the validity of the *Safety Zone* and the DTI-FT method for visualizing the OR.

### Conflict of interest statement

The authors declare that the research was conducted in the absence of any commercial or financial relationships that could be construed as a potential conflict of interest.

## Appendix

**Table A1:** **Results of the measurements performed before the dissection**.

**Brain 1 (in mm)**		**Brain 2 (in mm)**		**Brain 3 (in mm)**		**Brain 4 (in mm)**	
1r: 53	1l: 53	1r: 62	1l: 62	1r: 56	1l: 53	1r: 55	1l: 53
2r: 51	2l: 50	2r: 61	2l: 57	2r: 59	2l: 57	2r: 57	2l: 58
3r: 58	3l: 56	3r: 58	3l: 57	3r: 65	3l: 61	3r: 63	3l: 64
4r: 59	4l: 54	4r: 50	4l: 51	4r: 54	4l: 51	4r: 53	4l: 54
5r: 81	5l: 86	5r 99	5l: 98	5r: 97	5l: 100	5r: 99	5l: 104
6r: 129	6l: 129	6r: 127	6l: 123	6r: 133	6l: 132	6r: 135	6l: 131

*1, rostral part of brain stem to superior temporal gyrus; 2, caudal part of brain stem to middle temporal gyrus; 3, splenium of the corpus callosum to temporal cortex; 4, 25 mm caudal of splenium of the corpus callosum to temporal cortex; 5, parietal cortex to floor of the brain; 6, temporal pole to occipital pole. r, right hemisphere; l, left hemisphere*.

**Table A2:** **Results of the measurements performed after dissection**.

**Brain 1 (in mm)**		**Brain 2 (in mm)**		**Brain 3 (in mm)**		**Brain 4 (in mm)**	
7d: 26	7s: 25	7d: 35	7s: 34	7d: 30	7s: 28	7d: 27	7s: 25
8d: 29	8s: 30	8d: 40	8s: 37	8d: 36	8s: 35	8d: 34	8s: 37
9d: 30	9s: 36	9d: 34	9s: 36	9d: 42	9s: 39	9d: 43	9s: 41
10d: 33	10s: 31	10d: 28	10s: 30	10d: 34	10s: 32	10d: 33	10s: 33
11d: 68	11s: 72	11d: 83	11s: 85	11d: 82	11s: 86	11d: 86	11s: 90
12d: 100	12s: 102	12d: 103	12s: 100	12d: 107	12s: 113	12d: 107	12s: 104
Length OR: 103	Length OR:104	Length OR:104	Length OR:102	Length OR: 108	Length OR: 114	Length OR: 110	Length OR: 107

*7, rostral part of brain stem to OR; 8, caudal part of brain stem to outer border OR; 9, splenium of the corpus callosum to outer border OR; 10, 25 mm caudal of splenium of the corpus callosum to outer border OR; 11, parietal cortex to floor OR; 12, occipital pole to anterior part of the OR (Meyer's loop). r, right hemisphere, l, left hemisphere*.

**Table A3:** **Results of measurements performed before and after the dissection**.

**Brain 1 (in mm)**		**Brain 2 (in mm)**		**Brain 3 (in mm)**		**Brain 4 (in mm)**	
Ir (=1r-7d): 27	Il (=1l-7s): 28	Ir (=1r-7d): 28	Il (=ll-7s): 28	Ir(=1r-7d): 26	Il(=1l-7s): 25	Ir(=1r-7d): 28	Il(=1l-7s): 28
IIr (=2r-8d): 22	IIl (=2l-8s): 20	IIr (=2r-8d): 21	IIl (=2l-8s): 20	IIr(=2r-8d): 23	IIl(=2l-8s): 22	IIr(=2r-8d): 23	IIl(=2l-8s): 21
IIIr (=3r-9d): 28	IIIl (=3l-9s): 20	IIIr (=3r-9d): 24	IIIl (=3l-9s): 21	IIIr(=3r-9d): 23	IIIl(=3l-9s): 22	IIIr(=3r-9d): 20	IIIl(=3l-9s): 23
IVr (=4r-10d): 26	IVl (=4l-10s): 23	IVr (=4r-10d): 22	IVl (=4l-10s): 21	IVr(=4r-10d): 20	IVl(=4l-10s): 19	IVr(=4r-10d): 20	IVl(=4l-10s): 21
Vr (=5r-11d): 13	Vl (=5l-11s): 14	Vr (=5r-11d): 16	Vl (=5l-11s): 13	Vr(=5r-11d): 20	Vl(=5l-11s): 14	Vr(=5r-11d): 13	Vl(=5l-11s): 14
VIr (=6r-12d): 29	VIl (=6l-12s): 27	VIr (=6r-12d): 24	VIl (=6l-12s): 23	VIr(=6r-12d): 25	VIl(=6l-12s): 19	VIr(=6r-12d): 28	VIl(=6l-12s): 27
Length OR: 103	Length OR:104	Length OR:104	Length OR:102	Length OR: 108	Length OR: 114	Length OR: 110	Length OR: 107

*I, OR to superior temporal gyrus; II, OR to middle temporal gyrus; III, OR (at splenium corpus callosum) to temporal cortex; IV, OR (25 mm caudal of splenium of the corpus callosum) to temporal cortex; V, caudal part of OR to caudal part of inferior temporal gyrus; VI, temporal pole to Meyer's loop of OR. d, right hemisphere; l, left hemisphere*.

**Table A4:** **The mean average with standard deviation derived from the results of Table A3**.

**Mean average; all hemispheres (mm)**	**Standard deviation; all hemispheres (mm)**
*I*: 27.25	± 1.1
*II*: 21.5	± 0.97
*III*: 22.63	± 2.43
*IV*: 21.5	± 2.05
*V*: 14.63	± 2.23
*VI*: 25.25	± 3.07
Length OR: 106.5	± 3.8

*I, OR to superior temporal gyrus; II, OR to middle temporal gyrus; III, OR (at splenium corpus callosum) to temporal cortex; IV, OR (25 mm caudal of splenium of the corpus callosum) to temporal cortex; V, caudal part of OR to caudal part of inferior temporal gyrus; VI, temporal pole to Meyer's loop of OR*.

**Table A5:** **Lateral extension of the Optic radiations – dissection**.

	**Brain 1 (mm)**		**Brain 2 (mm)**		**Brain 3 (mm)**		**Brain 4 (mm)**	
**Location**	**R**	**L**	**R**	**L**	**R**	**L**	**R**	**L**
Temporal horn	14	16	15	14	18	17	19	19
Trigonum	20	21	23	22	25	23	25	25
Occipital horn	24	25	25	23	27	26	26	27
	**Mean average (mm)**				**Standard deviation (mm)**			
Temporal horn	16.5				± 1.84			
Trigonum	23				± 1.80			
Occipital horn	25.38				± 0.98			

*The lateral extension of the OR was measured at the temporal horn, trigonum, and occipital horn of the ventricle system. These locations correspond with the points 8, 9, and 10, respectively as is shown in Figure 5. R, right hemisphere; L, left hemisphere*.

**Table A6:** **Lateral extension of the Optic Radiations – DTI-FT**.

	**Brain 2 (in mm)**		**Brain 3 (in mm)**		**Brain 4 (in mm)**	
**Location of measurement**	**R**	**L**	**R**	**L**	**R**	**L**
Temporal horn	16.84	15.62	14.59	11.68	16.76	18.12
Trigonum of ventricles	22.98	21.33	20.91	20.18	21.23	20.53
Occipital horn	25.35	24.08	24.98	23.70	27.27	26.05
Length OR	107.08	106.80	101.98	103.20	101.95	114.36
	**Mean average (mm)**			**Standard deviation (mm)**		
Temporal horn	15.60			± 2.07		
Trigonum	21.19			± 0.89		
Occipital horn	25.24			± 1.20		
Length OR	105.90			± 4.32		

*The lateral extension of the OR was measured at the temporal horn, trigonum and occipital horn of the ventricle system. These locations correspond with the points 8, 9, and 10, respectively as is shown in Figure 4. D, right hemisphere; S, left hemisphere*.
